# Visualizing changes in electron distribution in coupled chains of cytochrome *bc*_1_ by modifying barrier for electron transfer between the FeS cluster and heme *c*_1_

**DOI:** 10.1016/j.bbabio.2009.11.003

**Published:** 2010-02

**Authors:** Ewelina Cieluch, Krzysztof Pietryga, Marcin Sarewicz, Artur Osyczka

**Affiliations:** Department of Biophysics, Faculty of Biochemistry, Biophysics and Biotechnology, Jagiellonian University, ul. Gronostajowa 7, 30-307 Kraków, Poland

**Keywords:** *E*_h_, ambient redox potential, *E*_m_, redox midpoint potential, *E*_m7_, redox midpoint potential at pH 7, *E*_m9_, redox midpoint potential at pH 9, FeS, 2 iron–2 sulfur cluster, Q, ubiquinone, QH_2_, ubihydroquinone, *Rba*., *Rhodobacter*, WT, wild type, Electron transfer, Cytochrome *bc*_1_, Complex III, Q cycle, Redox midpoint potential, Kinetic model

## Abstract

Cytochrome *c*_1_ of *Rhodobacter* (*Rba*.) species provides a series of mutants which change barriers for electron transfer through the cofactor chains of cytochrome *bc*_1_ by modifying heme *c*_1_ redox midpoint potential. Analysis of post-flash electron distribution in such systems can provide useful information about the contribution of individual reactions to the overall electron flow. In *Rba. capsulatus*, the non-functional low-potential forms of cytochrome *c*_1_ which are devoid of the disulfide bond naturally present in this protein revert spontaneously by introducing a second-site suppression (mutation A181T) that brings the potential of heme *c*_1_ back to the functionally high levels, yet maintains it some 100 mV lower from the native value. Here we report that the disulfide and the mutation A181T can coexist in one protein but the mutation exerts a dominant effect on the redox properties of heme *c*_1_ and the potential remains at the same lower value as in the disulfide-free form. This establishes effective means to modify a barrier for electron transfer between the FeS cluster and heme *c*_1_ without breaking disulfide. A comparison of the flash-induced electron transfers in native and mutated cytochrome *bc*_1_ revealed significant differences in the post-flash equilibrium distribution of electrons only when the connection of the chains with the quinone pool was interrupted at the level of either of the catalytic sites by the use of specific inhibitors, antimycin or myxothiazol. In the non-inhibited system no such differences were observed. We explain the results using a kinetic model in which a shift in the equilibrium of one reaction influences the equilibrium of all remaining reactions in the cofactor chains. It follows a rather simple description in which the direction of electron flow through the coupled chains of cytochrome *bc*_1_ exclusively depends on the rates of all reversible partial reactions, including the Q/QH_2_ exchange rate to/from the catalytic sites.

## 1 Introduction

Bioenergetic enzymes use chains of cofactors to transfer electrons over long distances and connect catalytic sites with substrate redox pools. In such systems, the overall direction of electron flow is a resultant of reversible partial reactions. When one, linearly organized chain is involved and the system behaves in purely electrochemical manner without any gating, the distribution of electrons among cofactors is relatively simple to predict based on equilibrium thermodynamic calculations. However complications arise when more than one chain is involved and the electron flow in one chain is somehow linked with the electron flow in the other chain or the group of chains. One of the prominent examples of this sort of arrangement is a two-chain assembly in cytochrome *bc*_1_ (complex III), a common component of respiratory and photosynthetic electron transfer systems.

In this enzyme a c-chain comprising high-potential cytochromes *c*_1_ and *c* and the 2 iron–2 sulfur cluster (FeS) and the b-chain comprising two low-potential hemes *b* (*b*_L_ and *b*_H_) and quinone of the Q_i_ site are linked together by the action of the centrally located quinone binding Q_o_ site. This site catalyzes a reversible two-electron oxidation of hydroquinone with one electron delivered into the c-chain and the other electron delivered into the b-chain. This is an integral part of the Q cycle used by the enzyme to catalyze electron transfer between quinone and cytochrome *c* [1–3].

Despite intense studies, our knowledge about the mechanism of the Q_o_ site catalysis and the way the two chains are connected is still not complete and thus remains a subject of intense debate. For the Q_o_ site reaction, several variations of either sequential or concerted mechanisms are considered [4–9]. For the chain connection, a thermodynamic coupling without any gating, or a coupling with various combinations of long-range allosteric interactions between the chains are considered [4–8,10–15]. The latter ones often assume some level of control over the large-scale motion of the FeS head domain, which during the catalytic cycle moves between the states where the FeS cluster approaches the Q_o_ site and the states where it approaches heme *c*_1_ [16]. Additional complications come from the observation that this enzyme functions as a dimer, and various types of intra-dimer interactions are also included in many of the proposed mechanisms [10,11,13,17,18]. The whole debate is intensified by the observation that under certain conditions cytochrome *bc*_1_ becomes vulnerable to superoxide production. This is considered to be a side reaction of the Q cycle with the most commonly accepted view that electrons leak on oxygen from a semiquinone formed in the Q_o_ site [4,19–22]. How this occurs is not known, but highly desired to be known as it is expected to improve our knowledge about the mechanism of the Q_o_ site action and to clarify some of the medically-related issues of radical production in living cells.

A convenient way to access experimentally certain electron transfer reactions of cytochrome *bc*_1_ is provided by membranes of purple photosynthetic bacteria where cytochrome *bc*_1_ can be light-activated through the allied photosynthetic reaction center. The electron flow through the two chains of cytochrome *bc*_1_ can be specifically manipulated by the use of inhibitors of catalytic Q_o_ or Q_i_ sites in combination with various site-directed mutations that purposely change some of the properties of cofactors [23]. Of those, mutations that target cytochrome *c*_1_ of *Rhodobacter* species turned out to be particularly useful, as they provided a whole series of variants which suitably alter electron transfer by modifying values of heme *c*_1_ midpoint potential [24–27].

**Fig. 1:**
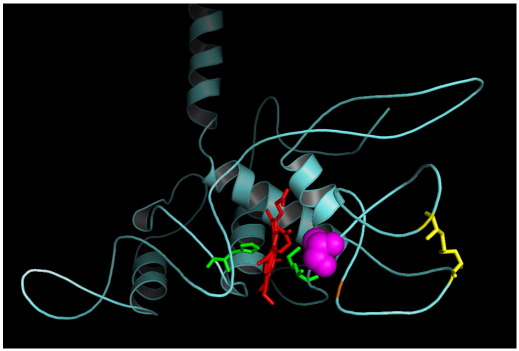
Ribbon model of the crystal structure of the heme domain of cytochrome *c*_1_ subunit of *Rba. capsulatus* cytochrome *bc*_1_[28]. Heme *c*_1_ (red sticks) is axially ligated by histidine and methionine (greens sticks). Position A181 (magenta spheres) is located within the sixth axial ligand domain. Position Y152 (orange line) is located between the two cysteine residues that form disulfide bond (yellow sticks).

One group of mutants includes the forms with mutations changing the sixth axial ligand to heme iron. In this case, an unusual tolerance of cytochrome *c*_1_ of *Rba. sphaeroides* to the alternations in the heme ligation pattern made it possible to obtain a whole range of mutants with modified potential of heme *c*_1_ which exposed the ultimate thermodynamic limits of operation of the cofactor chains in cytochrome *bc*_1_ [27]. The other group of mutants includes the low-potential forms of closely related *Rba. capsulatus* cytochrome *c*_1_ which are devoid of the disulfide bond naturally present in this species [26,28] (see Fig. 1) and in *Rba. sphaeroides* [29]. These non-functional forms revert spontaneously by introducing a second-site suppression (mutation A181T) that brings the potential of heme *c*_1_ back to the functionally high level yet maintains it some 100 mV lower from the native value. The revertants leave the disulfide bridge unrepaired [26].

In this work we report on some of the structural and redox properties of the mutant of cytochrome *c*_1_ of *Rba. capsulatus* in which the mutation A181T was introduced to the native protein containing all cysteine residues. We observe that the disulfide and A181T can coexist, but the mutation exerts a dominant effect on the redox properties of heme *c*_1_ and the potential remains at the same lower value as in the disulfide-free form. This established effective means to modify a barrier for electron transfer between the FeS cluster and heme *c*_1_ without breaking disulfide or changing the ligand to heme iron. We compared the flash-induced electron transfer in native and mutated cytochrome *bc*_1_ and found significant differences in the post-flash equilibrium distribution of electrons in the c-chain only when the connection of the chains with the quinone pool was interrupted at the level of either the Q_o_ or the Q_i_ site. To explain the results we developed a kinetic model which in our view improves the general understanding of electron flow through the coupled chains of cytochrome *bc*_1_.

## 2 Materials and methods

### 2.1 Bacterial strains, plasmids, and general molecular genetic techniques

*Rba. capsulatus* strains and *E. coli* (HB101) were grown in MPYE (mineral-peptone-yeast-extract) and LB media, respectively, supplemented with appropriate antibiotics as needed [30]. Photosynthetic growth of *Rba. capsulatus* strains was tested on MPYE plates using anaerobic jars (GasPak System, BBL) at 30 °C under continuous light. *Rba. capsulatus* strains with mutated cytochrome *bc*_1_ were generated as described previously [31], using the genetic system which was a generous gift of Prof. F. Daldal (University of Pennsylvania, Philadelphia, USA). The mutations A181T and A181T/Y152R were constructed by site directed mutagenesis. A 0.51 kb *Sph*I/*Sfu*I fragment of *pet*C gene was amplified by PCR with the set of two primers ASUII-F: 5′-CTTCAGC*TTCGAA*GGGATCTTCG (*Sfu*I site is shown in italic) and A181T-R: 5′-GG*GCATGC*GCG**T**CCAGCTGC (point mutation is shown in bold, *Sph*I site is in italic). The template DNA for PCR was either pPET1 [31] or pPET1-cY152R [26], a derivative of pBR322 containing either a wild-type copy of *pet*ABC or a copy of *pet*ABC with a single mutation Y152R in *pet*C, respectively. The resulting 0.51 kb PCR products containing either A181T or A181T/Y152R in *pet*C were first exchanged with their wild-type counterparts in pPET1 using the restriction enzymes *Sph*I/*Sfu*I and then inserted into pMTS1 (the expression vector containing a copy of *pet*ABC) using the restriction enzymes *Sfu*I/*Hind*III. The entire *pet*C gene was sequenced to ensure that the constructs contained the desired mutation and no other mutations. The mutated variants of pMTS1 were introduced into MT-RBC1 strain (*pet*ABC-operon deletion background) via triparental crosses as described in ref. [31]. In each case, the presence of engineered mutations was confirmed by sequencing the plasmid DNA isolated from the mutated *Rba. capsulatus* strains.

### 2.2 Isolation of cytochrome *bc*_*1*_ and electrophoresis

The chromatophore membranes were prepared from semiaerobically grown cultures of *Rba. capsulatus* as described previously [31,32]. Membranes were solubilized with dodecyl maltoside and the purification of cytochrome *bc*_1_ by the ion-exchange chromatography was performed as described in ref. [32]. Sodium dodecyl sulfate polyacrylamide gel electrophoresis (SDS-PAGE) was performed as described in ref. [26]. Samples of protein were incubated under reducing conditions at 60 °C for 5 min prior to the loading on gels (15% linear separating gel was used) and the gels were stained with Coomassie blue. The presence of disulfide bridge in cytochrome *c*_1_ containing A181T was verified using the factor Xa cleavage assay as descried in detail in ref. [26] on the form that contained the cleavage site introduced by mutation Y152R.

### 2.3 Optical spectroscopy and redox potentiometry

Optical spectra for *b*- and *c*-type cytochromes were recorded using Shimadzu UV-2450 spectrophotometer. The difference spectra were obtained with samples that were first oxidized by an addition of a potassium ferricyanide (to a final concentration of 20 μM) and then reduced by using either sodium ascorbate (added to a final concentration of 2 mM) or a minimal amount of solid sodium dithionite. Chemical oxidation–reduction midpoint potentials titrations of hemes *c* in membranes or purified complexes were performed as described in ref. [33]. The titrations were performed in 50 mM MOPS (for pH 7), 50 mM Tris (for pH 9) containing 100 mM KCl, and redox mediators: tetrachlorohydroquinone, 2,3,5,6-tetramethyl phenylenediamine, 1,2-naphthoquinone-4-sulfonate, 1,2-naphthoquinone, phenazine methosulfate, phenazine ethosulfate, and duroquinone at concentration of 5–15 μM. The optical changes that accompanied redox potential change were recorded in the α-region (500–600 nm) and the redox midpoint potentials (*E*_m_) of hemes *c* were determined by fitting the 552–542 nm difference to an *n* = 1 Nernst equation with one or two components, for the measurements with isolated complexes or membranes, respectively.

### 2.4 Flash-induced electron transfer measurements

Flash-activated turnover kinetics of cytochrome *bc*_1_ were performed essentially as described in refs. [34–36], on a home-built double wavelength time-resolved spectrophotometer. The spectrophotometer consisted of a SP-20 Flash Lamp System (Rapp OptoElectronic, GmbH) and an optical assembly equipped with two H-20Vis monochromators (JY Horiba) (a generous gift of Prof. P. Leslie Dutton, University of Pennsylvania, Philadelphia, USA) and the two 9828SB07 photomultipliers in QL30 housing (Electron Tubes). An activation of the sample and an acquisition of the data were controlled by a locally written program using the NI PXI-1042Q interface (National Instruments). The electronic assembly was designed by T. Oleś and J. Kozioł (Jagiellonian University). For the measurements, chromatophore membranes were suspended in appropriate buffer: 50 mM MES (for pH 6), 50 mM MOPS (for pH 7), or 50 mM Tris (for pH 9) containing 100 mM KCl, 3.5 μM valinomycin, and redox mediators: 7 μM 2,3,5,6-tetramethyl-1,4-phenylenediamine, 1 μM phenazine methosulfate, 1 μM phenazine ethosulfate, 5.5 μM 1,2-naphthoquinone, 5.5 μM 2-hydroxy-1,4-naphthoquinone. The sample was poised at an ambient potential of 150 mV, 100 mV, or -20 mV for pH 6, 7, or 9, respectively. Transient cytochrome *c* and *b* reduction kinetics initiated by a short saturating flash (10 μs) from a xenon lamp were followed at 550–540 nm and 560–570 nm, respectively. Inhibitors antimycin A, myxothiazol, and stigmatellin were used at final concentrations of 7, 7 and 1.5 μM, respectively.

### 2.5 Kinetic model of flash-activated electron transfer

Simulation of flash-activated curves were performed using XPPAUT 5.85 [37] included in Ubuntu Linux 8.10. The model was built from a set of 28 differential equations, from which 24 describe the changes in the concentrations of particular states of cytochrome *bc*_1_ monomer (i.e., redox states of cofactors, occupancy of Q_o_ site, position of the FeS head domain) and the remaining four equations describe the changes in the concentrations of substrates (i.e., quinone (Q), quinol (QH_2_) and cytochrome *c*). The equations were constructed taking into account the following assumptions: (1) molar ratio of total quinone to cytochrome *bc*_1_ monomer is 20:1, (2) single flash generates two oxidized molecules of cytochrome *c* per cytochrome *bc*_1_ monomer [38], (3) affinities of Q and QH_2_ to the Q_o_ are equal, (4) all steps are reversible, (5) all reactions within a monomer are independent on each other and each monomer can work independently, (6) reduction of Q and oxidation of QH_2_ at the Q_o_ site are both concerted. The individual reactions in the high-potential c-chain were taken for detailed calculations. The b-chain, for simplicity, was described by one equation, in which the presence or absence of inhibitors was expressed by variations in the time constants describing concerted electron transfer from QH_2_ to FeS and heme *b*_L_ or from heme *b*_L_ and FeS to Q at the Q_o_ site. At the beginning of the simulations the concentrations of Q and QH_2_ were the same and all Q_o_ sites of the cytochrome *bc*_1_ were occupied. The effect of change in the difference of potential between the FeS center and heme *c*_1_ was included in the time constants for electron transfer rates between FeS and heme *c*_1_ estimated using the equations of Moser and Dutton [39,40]. In these calculations, the distance of 11.4 Å between cofactors and reorganization energy of 0.7 eV were taken [41]. All remaining rate constants were adjusted to correctly reproduce the experimental curves. The simulated curves were obtained by numerical integration of the differential equations with XPPAUT using Gear's algorithm and the outputs were analyzed using home-written LabView program. The XPPAUT script used for simulations is included in Supplementary Material.

## 3 Results and discussion

### 3.1 Structural and redox properties of the family of A181T (βXM) mutants

Earlier work has established two independent structural motifs that contribute to controlling the integrity of the heme binding pocket to raise the redox potential in cytochrome *c*_1_ of *Rba. capsulatus* [26]. The native protein employs, an uncommon for cytochromes *c*, disulfide anchoring of the extra loop (C144-C167) adjacent to the domain of the sixth axial ligand of heme iron (M183) (Fig. 1). The engineered disulfide-free protein (containing cysteine to alanine mutations at positions 144 and 167) has a loosened structure with the redox midpoint potential lowered to nonfunctional levels unless an additional suppressor mutation is present in the form of a β-branched amino acid two residues away from the heme-ligating methionine (A181T) (Fig. 1). With this mutation (considered as a part of a so called βXM motif, naturally present in many other cytochromes *c*) cytochrome *c*_1_ reaches back a functional range of high redox potential (*E*_m7_ = 227 mV). The value of the potential is, however, still lower by 100 mV from the value of native cytochrome *c*_1_ [26].

**Fig. 2:**
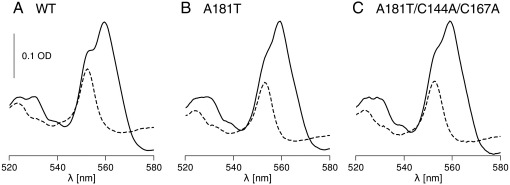
Optical redox difference spectra of cytochrome *bc*_1_ isolated from wild type (A), A181T mutant (B) and A181T/C144A/C167A mutant (C). Solid and dashed lines correspond to dithionite minus ferricyanide and ascorbate minus ferricyanide spectra, respectively.

**Fig. 3:**
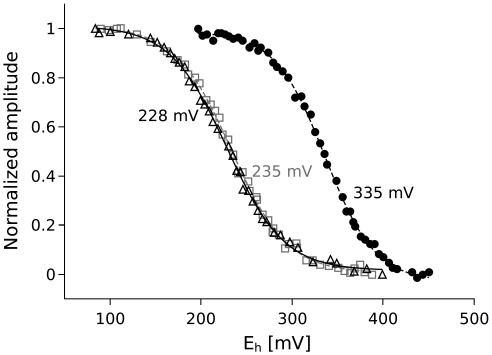
Potentiometric dark equilibrium titration of heme *c*_1_ in isolated wild-type cytochrome *bc*_1_ (closed black circles), A181T mutant (open grey squares) and A181T/C144A/C167A triple mutant (open black triangles). The titrations were performed at pH 7, and experimental data were fit to the Nernst equation for a one-electron couple. The *E*_m_ values obtained are shown in the figure and also listed in Table 1.

To test the effect of the presence of the βXM motif alone, we introduced A181T to the protein in which the cysteines at positions 144 and 167 were not changed. With this mutation, cytochrome *bc*_1_ is functional in vivo (PS+ phenotype) (Table 1) and the optical difference spectra of purified complexes have the ascorbate-reducible component at 552 nm reminiscent of the presence of high potential cytochrome *c* (Fig. 2, dashed line). The shape of the spectra of cytochrome *bc*_1_ fully reduced with dithionite (Fig. 2, solid line) immediately suggests some similarities between A181T and A181T/disulfide-free cytochrome *c*_1_: in both spectra the 552 nm component of cytochrome *c*_1_ is less separated, in comparison to the wild type, from the component at 560 nm corresponding to cytochromes *b*. Even more significantly, the redox midpoint potential of A181T cytochrome *c*_1_ has a very close value to that of A181T/disulfide-free cytochrome *c*_1_ (235 vs. 228 mV) which, again, is about 100 mV lower than in the wild-type (Table 1, Fig. 3).

This apparently dominant effect of mutation A181T on the redox properties of cytochrome *c*_1_ raises an interesting structural question as to how much the specific orientation of the loop regions reinforced by a reduced conformational flexibility of T181 [26] differs from the native structure. The most severe case would feature A181T not allowing the disulfide to form even with C144 and C167 present. To test this, we used a factor Xa cleavage assay (described in detail in ref. [26]) on a variant of A181T which contained a factor Xa cleavage site in the region between C144 and C167 (i.e., had additional mutation Y152R, see Fig. 1). The results (Fig. S1 in Supplementary Material) revealed that a disulfide bond does form in A181T/Y152R (and by extrapolation in A181T) indicating that the A181T mutation and the disulfide can coexist in one protein (Table 1). Thus, to explain the very same drop in the redox potential of both of A181T mutants (i.e., in disulfide-free and disulfide-containing form), one may speculate that those mutants adopt similar overall conformation which somehow differs from the native conformation, yet features the loop regions oriented in such a manner that disulfide bond is formable provided that C144 and C167 are present (i.e., the alanines at positions 144 and 167 in disulfide-free A181T mutant are in approximately the same orientation as the cysteines 144 and 167 in disulfide-containing A181T).

To what extent the A181T mutants differ structurally from the native cytochrome *c*_1_ and how this relates to the observed change in the redox potential requires further studies which are beyond the scope of this work. We focused on using the redox properties of A181T mutants as the effective means to unravel some of the mechanistic elements of electron transfer within the cofactor chains of cytochrome *bc*_1_. For this purpose we chose A181T mutant containing disulfide bond. Even though, as discussed earlier, all A181T mutants might adopt similar overall conformation, the possibility that it more resembles the native structure (i.e., structural “side effects” are minimized) is always higher in the disulfide-containing rather than disulfide-free form.

### 3.2 Light-induced electron transfer in wild type and A181T

**Fig. 4:**
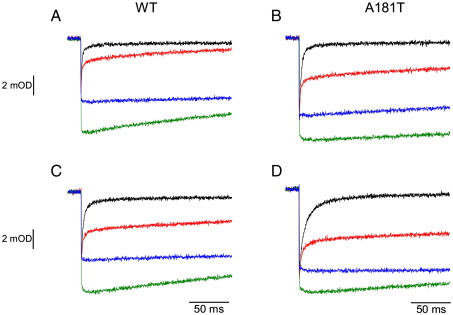
Flash-activated cytochrome *c* oxidation and re-reduction in chromatophores containing wild-type cytochrome *bc*_1_ (A, C) and the A181T mutant (B, D). Kinetic transients at 550–540 nm were recorded at pH 7 (A, B) or pH 6 (C, D) with the Q pool half-reduced, in the absence of inhibitors (black) and in the presence of antimycin (red), myxothiazol (blue) or stigmatellin (green).

Fig. 4 compares kinetic transients of electron transfer in the c-chain of wild type cytochrome *bc*_1_ with those of A181T mutant. The characteristic and well-known traces recorded for the wild type at pH 7 and ambient potential of 100 mV (which maintains the Q pool half-reduced) include: the trace for the non-inhibited system, where the light-induced oxidation of cytochromes *c* is followed by a complete re-reduction phase (Fig. 4A, black); the trace with the Q_i_ inhibitor antimycin present where cytochrome *c* re-reduction occurs but on the millisecond timescale is not completed (Fig. 4A, red); and the traces with two specific inhibitors of the Q_o_ site, stigmatellin or myxothiazol (Fig. 4A, green or blue, respectively). The trace with stigmatellin reveals the full extent of flash-oxidized cytochrome *c* (in this case the FeS cluster is locked at the Q_o_ site due to interactions with inhibitor and can not interact directly with cytochrome *c*_1_), while the trace with myxothiazol reveals the amount of cytochrome *c* that remains oxidized after initial reduction by the pre-reduced FeS cluster (in this case the FeS cluster is not locked at the Q_o_ site and can interact with cytochrome *c*_1_); see description of myxothiazol and stigmatellin traces in ref. [42]. The rate of reduction of cytochrome *c* by the pre-reduced FeS cluster is faster than the time resolution of the method, thus in the presence of myxothiazol we only register the final level of oxidized cytochrome *c* which gives a smaller amplitude than the amplitude of oxidized cytochrome *c* recorded in the presence of stigmatellin. The corresponding traces of heme *b* oxidation/reduction are not shown; only the rate of heme *b* reduction in the presence of antimycin is reported in Table 2.

In the next parts, the difference in the level of the oxidized cytochrome *c* recorded in the presence of stigmatellin and the level of cytochrome *c* re-reduced in the presence of antimycin will be referred as “antimycin phase,” and the difference between the level of oxidized cytochrome *c* in the presence of stigmatellin and that recorded in the presence of myxothiazol will be referred as “myxothiazol phase.”^1^ These two phases reveal major differences between the wild type and the A181T mutant.

In A181T under the same experimental conditions (pH 7, *E*_h_ 100 mV) the re-reduction of cytochrome *c* in the absence of any inhibitors is slower but complete (Fig. 4B, black, see Table 2 for corresponding rate of heme *b* reduction measured in the presence of antimycin). Remarkably, the antimycin phase has significantly lower amplitude than that of wild-type (less cytochrome *c* gets re-reduced in the presence of antimycin) (Fig. 4B, Table 3). Also the myxothiazol phase is smaller (in the mutant it is approximately one fourth of the total amplitude of flash-oxidized cytochrome *c* seen in the presence of stigmatellin, while in the wild-type it is approximately one third of the total amplitude) (Fig. 4B, Table 3).

At acidic pH (pH 6, *E*_h_ 150 mV maintaining the Q pool half-reduced), the antimycin phase in the wild type and the mutant (Fig. 4C, D; Table 3) is smaller comparing to the respective phases recorded at pH 7. The myxothiazol phase remains similar in the wild type, while it slightly diminishes in the mutant (Fig. 4C, D; Table 3), when again compared to the respective phases recorded at pH 7. At alkaline pH (pH 9, *E*_h_ -20 mV) the antimycin phase much increases both in the wild type and the mutant, approaching the level of fully re-reduced cytochrome *c* in the absence of inhibitors (Table 3). The myxothiazol phase in both cases also increases to reach about a half of the total amplitude of flash-oxidized cytochrome *c* seen in the presence of stigmatellin (Table 3). At this pH the differences between the wild type and the mutant fade out.

With this kinetic screening over pH certain features become apparent. When the mutant is compared with the wild type at given pH, the changes in the post-flash equilibrium distribution of electrons are most prominent when antimycin is present (the antimycin phase is generally smaller in the mutant) but are not seen with the non-inhibited system (cytochrome *c* is fully re-reduced in both the wild type and the mutant). A similar type of change in the distribution of electrons is also generated by changing pH (the antimycin phase decreases with lowering pH in both the mutant and the wild type).

### 3.3 Barrier for electron transfer between the FeS cluster and heme *c*_1_

**Fig. 5:**
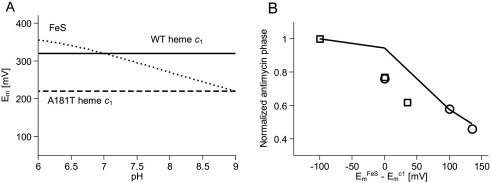
Difference in midpoint potential between the FeS cluster and heme *c*_1_ and its influence on the post-flash distribution of electrons in the c-chain in the presence of antimycin. (A) pH-dependence of *E*_m_ of the FeS cluster (dotted line) and heme *c*_1_ in wild type (solid line) and in A181T mutant (dashed line). Horizontal dashed line for A181T is assumed from the similar values of *E*_m_ measured at pH 7 and 9 (Table 1). (B) The experimentally determined fraction of cytochrome *c* re-reduced in the wild type (open squares) and A181T mutant (open circles) in the presence of antimycin (antimycin phase) is plotted vs. difference in *E*_m_s between the FeS cluster and heme *c*_1_. Solid line represents the changes in the fraction of cytochrome *c* re-reduced simulated for the conditions when antimycin is present by the model described in Fig. 6D.

Fig. 5A illustrates a shift in the *E*_m_ of heme *c*_1_ in A181T across pH and the effect of pH on the *E*_m_ of the FeS cluster. Since the *E*_m_ of heme *c*_1_ in A181T is 100 mV lower than that of wild type (Fig. 3, Table 1), it is reasonable to expect that changes in electron distribution, reminiscent of a barrier for electron transfer, are correlated with a difference in *E*_m_s between the FeS cluster and heme *c*_1_; the lower the *E*_m_ of heme *c*_1_ will be with respect to the *E*_m_ of the FeS cluster, the larger the barrier for electron transfer will be and consequently less of cytochrome *c* will get reduced in the flash experiment. This effect should strongly depend on pH, as the barrier of potential difference strongly depends on pH (in the tested pH range the *E*_m_ of FeS cluster falls with pH increase while that of heme *c*_1_ does not; Fig. 5A and ref. [43]). The about 100 mV barrier present at pH 7 (the *E*_m7_ of the FeS cluster is 320 mV) is much diminished at pH 9, where the *E*_m_ of the FeS cluster drops about 120 mV and approaches that of mutated cytochrome *c*_1_ (*E*_m9_ = 223 mV; Table 1). On the other hand, at pH 6 the *E*_m_ of the FeS cluster raises to about 350 mV, which increases the barrier present at pH 7. Indeed, the pH dependence of the amplitude of the antimycin phase appears to track the size of the barrier (the amplitude decreases with decreasing pH as the barrier increases) (Fig. 5B, circles).

With these considerations, a similar tracking becomes evident also for the wild type (Fig. 5B, squares). In this case the barrier of potential difference changes with pH in the same direction, although its absolute value at given pH is smaller comparing to the mutant (*E*_m_ of heme *c*_1_ is higher) (Fig. 5A). Consequently, the amplitude of the antimycin phase also decreases with decreasing pH, although its size at given pH (in particular at pH 6 and 7) is larger than the respective amplitude in the mutant (Fig. 4, Table 3).

The changes in the barrier of potential difference between the FeS cluster and heme *c*_1_ are also reflected in the changes in the amplitude of myxothiazol phase. This phase diminishes from about a half of the total amplitude of the oxidized cytochrome *c* at pH 9 (in both the wild type and the mutant), to about one third in wild type or one fourth in the mutant at pH 7 and 6 (Table 3). Not only this dependency can again be correlated with increase in the barrier of potential difference, but also the smaller myxothiazol phase in the mutant at pH 7 or 6 in comparison to the wild type appears to reflect a larger barrier of potential difference at these two pHs in the mutant.

It thus become clear that the barrier of the difference in midpoint potential between the FeS cluster and heme *c*_1_ does influence the post-flash distribution of electrons in the c-chain in the presence of antimycin or myxothiazol. Dropping a midpoint potential of heme *c*_1_ by mutation acts only to enhance the effects and trends naturally present in the native system. This, in principle, helps in interpreting the results. However, the electron exchange between the FeS cluster and heme *c*_1_ is only one step in the more complex reaction sequence involving two chains of cofactors. Thus understanding of the meaning of the changes in the post-flash distribution of electrons in one chain under the blockage of the outflow of electrons from the second chain (the antimycin case) requires modeling.

### 3.4 Kinetic model of electron flow in the c-chain

We consider the kinetic model which uses the postulates of the original equilibrium model described in ref. [6]: an adequacy of the equilibrium redox potential values, the amount of oxidized equivalents per single flash, and a strict coupling of reactions at the Q_o_ site. The latter postulate accommodates the Q/QH_2_ exchange at the Q_o_ site which means that the forward reaction takes place when the QH_2_, oxidized FeS and oxidized heme *b*_L_ are all present at the site, while the reverse reaction takes place when the Q, reduced FeS and reduced heme *b*_L_ are all present at the site. In this model all steps of electron transfer are reversible and independent of each other. In addition, the effect of change in distance between cofactors on the electron transfer coming from the movement of the FeS head domain is simplified to include just the two states that switch between themselves independently of any other event in the enzyme. Because all the experiments were done at *E*_h_ equal to the *E*_m_ of the quinone pool, the concentrations of Q and QH_2_ are considered equal.

**Fig. 6:**
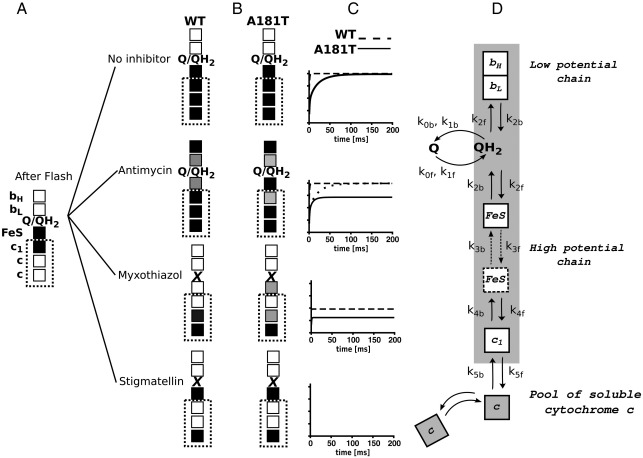
Analysis of post-flash electron distribution in wild type and A181T cytochrome *bc*_1_. Schemes in A, B compare wild type (WT) with A181T for a given set of conditions. Black and white squares represent reduced and oxidized cofactors, respectively. Incomplete reduction is represented in a grey scale. Dotted squares mark the cofactors of c-chain that are observable in the flash experiments. (A) Single flash generates two oxidizing equivalents per cytochrome *bc*_1_ (white squares *c*) [38] at the time where hemes *b* are oxidized (white squares *b*_L_ and *b*_H_) and the FeS cluster and heme *c*_1_ are reduced (black squares FeS and *c*_1_, respectively). (B) Within tens of milliseconds electrons redistribute to reach equilibrium in which the reduction levels of cofactors vary depending on experimental conditions. In the absence of inhibitors, unperturbed electron flow out of the b-chain upon oxidation of two QH_2_ secures complete re-reduction of all cofactors in the c-chain in both WT and A181T (black squares in no inhibitor panel). In the presence of antimycin, the level of reduced hemes *b* available for reverse reaction is higher and the equilibrium is reached before the c-chain is fully reduced. In WT, incomplete oxidation of second QH_2_ (grey squares in antimycin panel) leaves FeS partially oxidized, which leads to redistribution of electrons in the entire c-chain, as observed by flash at the level of cytochromes *c* (note that intensity of squares in c-chain should be reduced, which for simplicity is not shown). In A181T, incomplete oxidation of second QH_2_ faces additional barrier of potential difference between FeS and heme *c*_1_ which shifts equilibrium toward reduced FeS at the expense of reduced heme *c*_1_ (represented as black square FeS and light grey square *c*_1_). This increases probability of reverse reaction and decreases probability of forward reaction at the Q_o_ site, and the level of reduced heme *b*_L_ may decrease (note that in this case the reduction level of heme *c*_1_ determines the reduction level of heme *b*_L_, as represented by light grey squares *c*_1_ and *b*_L_). In the presence of myxothiazol (myxothiazol panel), electron from pre-reduced FeS cluster redistributes among the cofactors of the c-chain, but in WT the oxidation of FeS cluster by cytochromes *c* is more prominent than in A181T (note that for simplicity, for WT a complete electron transfer from FeS cluster to heme *c* is shown, white square FeS and black squares c). In the presence of stigmatellin (stigmatellin panel) only cytochromes *c* are in equilibrium thus full extent of flash-oxidized cytochromes *c* is preserved. Panel (C) shows simulations of the traces for the reduction of the observable experimentally cytochromes *c* in WT (dashed line) and A181T (solid line) obtained from the model schematically presented in panel (D). The time constants for partial reactions denote *k*_0f_/*k*_0b_ — association/dissociation rate of Q to/from the Q_o_ site, *k*_1f_/*k*_1b_ — same as *k*_0f_/*k*_0b_ but for QH_2_, *k*_2f_/*k*_2b_ — two-electron oxidation/reduction of QH_2_/Q in the Q_o_ site, *k*_3f_/*k*_3b_ — rate constants for movement of the FeS head domain to/from cytochrome *c*_1_ position, *k*_4f_/*k*_4b_ — rate constants for electron transfer from FeS to heme *c*_1_ or reverse reaction, *k*_5f_/*k*_5b_ — rate constant for electron transfer from heme *c*_1_ to heme *c* or reverse reaction. Dotted line in the antimycin panel in C shows the trace simulated for A181T assuming that QH_2_ oxidation at the Q_o_ site was concerted but irreversible (i.e., *k*_2b_ = 0 M^-1^s^-1^). This illustrates the prediction of how the system would respond if at least one reaction was irreversible (a case not supported experimentally).

Figs. 5B and 6 show that with these simple assumptions the model generally reproduces the observed trends and changes in the post-flash electron distribution in the wild type and the mutant, with and without inhibitors, at different pHs. This guides us into the following description of electron transfer reactions.

The full cytochrome *c* re-reduction in the absence of any inhibitor consumes electrons from the pre-reduced FeS cluster and then from quinols that are oxidized at the Q_o_ site in a coupled reaction that delivers the second quinol-born electron to the b-chain. A completeness of this reaction is a consequence of the unperturbed outflow of electrons from hemes *b* to the Q_i_ site and further down to the Q pool. In another words, a completion of two reactions of QH_2_ oxidation at the Q_o_ site secured by an immediate removal of electrons from hemes *b* leaves all cofactors in the c-chain (including the FeS cluster) fully saturated with electrons. The reaction is driven to completion even when heme *c*_1_ has potential 100 mV lower than *E*_m_ of FeS in the mutant (Fig. 6, no inhibitor panel). This is consistent with the observations that the *E*_m_ of heme *c*_1_ can be lowered within this range without affecting the overall electron flow through the c-chain [27,44]. In fact, the difference in *E*_m_s between heme *c*_1_ and the FeS cluster can be as much as 180 mV and the enzyme still muster enough electron transfer through cytochrome *bc*_1_ to support its functionality [27].

However, when the outflow of electrons from the b-chain is blocked with antimycin, the b-chain gets saturated with electrons and hemes *b* remain reduced for significant amount of time. This increases the effective concentration of one of the substrate needed for reverse reaction at the Q_o_ site (reduced heme *b*_L_). The presence of reduced heme *b*_L_ can shift the equilibrium of the Q_o_ site reaction depending on the availability of the other substrates for the reverse reaction (quinone and the reduced FeS cluster). It follows that, at a given Q concentration, a probability of the reverse reaction increases as the effective concentration of reduced FeS cluster at the site increases. This happens, for example, when the barrier for electron transfer from the FeS cluster to heme *c*_1_ increases, which in our experiments was achieved either by lowering the *E*_m_ of heme *c*_1_ by mutation or by increasing the *E*_m_ of the FeS cluster through the pH change. Once the oxidation of the second quinol cannot be completed, the c-chain cannot be fully saturated with electrons and consequently the equilibrium distribution of electrons in the entire c-chain is shifted (Fig. 6, antimycin panel). This means a partial backflow of electrons from cytochrome *c* reflected as a change between the level of reduced cytochrome *c* in the absence and the presence of antimycin. The model would thus predict that increasing the barrier for electron transfer from the FeS cluster to heme *c*_1_ would result in a decrease of the amplitude of the antimycin phase (Figs. 5B, 6C), which is consistent with the experiment (Figs. 4, 5B, Table 3). With this reasoning we can understand why affecting the equilibrium distribution of electrons in the b-chain in the presence of antimycin affects the distribution of electrons in the c-chain (see details in Fig. 6).

We note that if the reaction at the Q_o_ site was considered irreversible, the model would not predict shifts in the presence of antimycin and cytochrome *c* reduction would always run to completion (see Fig. 6C, dotted line). Similarly, a complete cytochrome *c* reduction would occur if the reduction of the FeS cluster at the Q_o_ site would be kinetically separated from its oxidation by cytochrome *c* (an inherent feature of many gating mechanisms). This clearly is not supported experimentally.

In the presence of myxothiazol, the situation is simplified to only include the post-flash equilibrium distribution of electron that comes from the pre-reduced FeS center. In this case the reduction level of cytochrome *c* again depends of the height of the barrier for electron transfer from the FeS cluster to heme *c*_1_. The model predicts that increasing the barrier decreases the myxothiazol phase (Fig. 6, myxothiazol panel) consistent with the experimental observations (Fig. 4, Table 3).

In conclusion, the variations in the distribution of electrons demonstrated in flash experiments guide us into an appreciation of a mechanism in which a shift in the equilibrium of one reaction influences the equilibrium of all remaining reactions in the cofactor chains. To allow this, all the reactions must be kinetically coupled and reversible on a millisecond timescale [6]. This means that, the FeS cluster, which undergoes a movement during catalysis, must equilibrate with both of its partners on a millisecond timescale independently of its redox state and of the redox state of the partners. This would be consistent with a recent observation about a presence of broad distribution of positions of FeS head domain in native cytochrome *bc*_1_ [45]. Mechanistically, this model does not require any physical gating, but at the same time it does not preclude it. This model follows a rather simple description in which the direction of electron flow through both of the chains exclusively depends on the rates of all reversible partial reactions, including the Q/QH_2_ exchange rate to/from the catalytic sites.

## Figures and Tables

**Table 1:** Effect of A181T mutation on redox properties of cytochrome *c*_1_ with and without the presence of the disulfide bridge.

	PS^a^	*E*_m7_ isolated^b^	*E*_m_ membranes^c^	Disulfide bridge
WT	+	335	328^(pH7)^	+
A181T	+	235	229^(pH7)^; 223^(pH9)^	+^d^
A181T/C144A/C167A	+	228	ND	-

a Photosynthetic phenotype; + indicates photosynthetic growth ability.

b Potential of heme *c*_1_ in isolated complexes.

c Potential of heme *c*_1_ in membranes where titrations show an additional component of + 377 mV (cytochromes *c*_2_ and *c*_y_); ^(pH7)^ and ^(pH9)^ indicate pH for which a value of *E*_m_ is given.

d Based on the results of the factor Xa cleavage assay performed with a form containing the factor Xa cleavage site (A181T/Y152R).

**Table 2:** Rates of flash-induced heme *b*_H_ reduction in the presence of antimycin (s^-1^).

pH	WT	A181T
6	566	386
7	1051	656
9	1791	1301

**Table 3:** Fraction of cytochrome *c* re-reduced in chromatophores containing wild-type cytochrome *bc*_1_ or the A181T mutant after its oxidation by single flash in the presence of antimycin or myxothiazol (antimycin or myxothiazol phase).

	WT			A181T		
	pH 6	pH 7	pH 9	pH 6	pH 7	pH 9
Antimycin phase	0.62	0.77	1	0.46	0.58	0.78
Myxothiazol phase	0.31	0.35	0.50	0.23	0.27	0.50

## Footnotes

1 In flash experiments, traces recorded in the presence of stigmatellin often show unspecific drift upwards (this drift becomes larger as pH increases), as most clearly seen in Fig. 4A. For each trace, the beginning of the linear part of the curve was taken to calculate the amplitude of antimycin and myxothiazol phase.
