# Supplementary material for: Visualizing changes in electron distribution in coupled chains of cytochrome bc1 by modifying barrier for electron transfer between the FeS cluster and heme c1
Source: Biochim Biophys Acta. 2010 Feb;1797(2):296–303. doi: 10.1016/j.bbabio.2009.11.003 (PMC2807467; doi:10.1016/j.bbabio.2009.11.003)
Supplement: Supplementary material [file mmc1.doc]

**SUPPLEMENTARY MATERIAL**

**Visualizing changes in electron distribution in coupled chains of cytochrome *bc*_1_ by modifying barrier for electron transfer between the FeS cluster and heme *c*_1_**

Ewelina Cieluch, Krzysztof Pietryga, Marcin Sarewicz, and Artur Osyczka

*Department of Biophysics, Faculty of Biochemistry, Biophysics and Biotehcnology,*

*Jagiellonian University, Kraków, Poland*


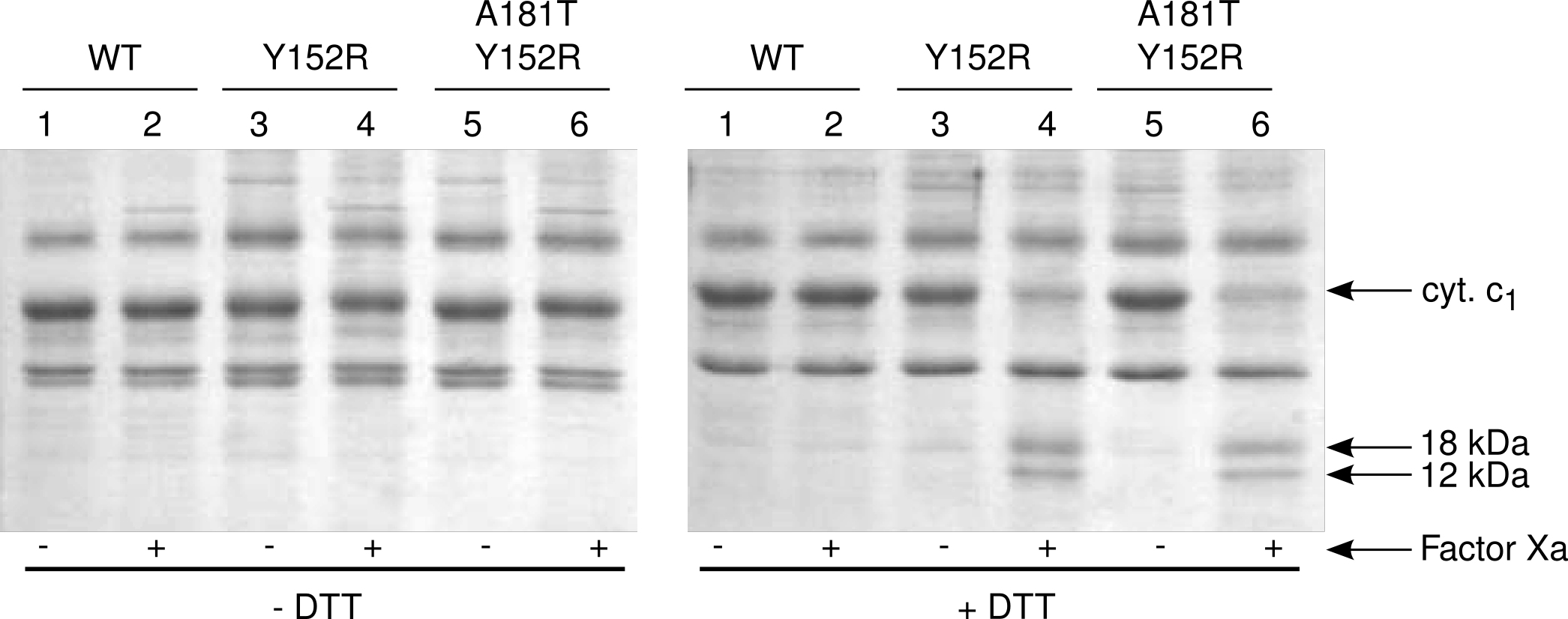


**Figure S1**. The test on the presence of disulfide bond in cytochrome *c*_1_ subunit, based on the method of proteolytic cleavage of the protein with Factor Xa. SDS-Page of cytochrome *bc*_1_ isolated from Wild-type, Y152R mutant and A181T/Y152R double mutant was carried out in the absence (-DTT) and presence of dithiotreitol (+ DTT). Prior to the electrophoresis samples were incubated in the buffer with (2,4,6) and without addition of Factor Xa (1,3,5).

**XPPAUT script used for simulations**

# XPPAUT input script of the kinetic model of cytochrome bc1

# (http://www.math.pitt.edu/~bard/xpp/xpp.html)

#---------------------------------------------------------------------------

# Rate constants:

# k0f - association rate const. of Q to Q_o site [1/microM*1/ms]

# k0b - dissociation rate cont. of Q from Q_o site [1/ms]

# k1f - association rate const. of QH_2 to Q_o site [1/microM*1/ms]

# k1b - dissociation rate const. of QH_2 from Q_o site [1/ms]

# k2f - rate const. of forward, concerted electron transfer from QH_2 to

# oxidized FeS and oxidized heme b_L [1/ms]

# k2b - rate const. of reverse, concerted electron transfer from reduced FeS

# and heme b_L to Q at Q_o site [1/ms]

# k3f - rate const. of FeS head domain movement from Q_o position toward c_1

# position [1/ms]

# k3b - rate const. of FeS head domain movement from c_1 position toward Q_o

# position [1/ms]

# k4f - rate const. of electron transfer from FeS cluster to heme c_1 [1/ms]

# k4b - rate const. of electron transfer from heme c_1 to FeS [1/ms]

# k5f - rate const. of electron transfer from heme c_1 to heme c_2 including

# association of cyt. c_2 to cytochrome bc_1 [1/microM*1/ms]

# k5b - rate const. of electron transfer from heme c_2 to heme c_1 including

# dissociation of cyt. c_2 from cytochrome bc_1 [1/ms]

# Initial parameter for the concentration of the states of the enzyme. Name of

# the each state includes information about occupancy of the Qo site,

# redox state of position of the FeS head domain and redox state of cytochrome

# c_1.

# (1) EmpXXXXX, (2) QoXXXXX, (3) QHoXXXXX - denotes the states with empty (2)

# Q_o site and occupied by Q (2) or QH_2 (3) [microM]

# (4) XXXFobXX, (5) XXXFocXX (6) XXXFrbXX, (7) XXXFrcXXX - denotes the states

# with oxidized FeS cluster at Q_o position (4) and c_1 position (5),

# and the respective positions of the reduced cluster (6) and (7). [microM]

# (8) XXXXXXCo, (9) XXXXXXCr - denotes the states with oxidized and reduced

# cytochrome c_1 [microM]

# (10) Cyt, (11) Cred, (12) - concentrations of oxidized (10) and

# reduced cytochrome c_2 [microM]

# (12) Q, (13) QH_2 - concentration of quinone and quinol respectively [microM]

#

# The respective concentrations of the all states at the beginning of the

# simulations were set assuming that no empty Q_o sites are present

# and the Q/QH_2 occupy the equal number of Q_o sites. Fraction of

# reduced/oxidized cofactors at given Eh and pH were calculated using Nernst

# eq.

# EXAMPLE OF Initial conditions used for simulations

init QoFobCo=0, QoFobCr=0,QoFocCo=0, QoFocCr=0,QoFrbCo=0, QoFrbCr=0.25,QoFrcCo=0, QoFrcCr=0.25,QHoFobCo=0,QHoFobCr=0,QHoFocCo=0,QHoFocCr=0, QHoFrbCo=0,QHoFrbCr=0.25, QHoFrcCo=0,QHoFrcCr=0.25,EmpFobCo=0,EmpFobCr=0,EmpFocCo=0,EmpFocCr=0,EmpFrbCo=0,EmpFrbCr=0,EmpFrcCo=0,EmpFrcCr=0

init QH2=9.5, Q=9.5, CYT=2, CRED=0

################# P A R A M E T E R S E T T I N G S ######################

# You must remove the # mark from the begining of the line that describes the

# parameters determining the particular case.

# pH = 9, Eh = -20 mV

# ---------------- non-inhibited cytochrome bc1 ----------------------

# par k0f=4, k0b=4, k1f=4, k1b=4, k5f=200, k5b=0.3, k2f=60, k2b=0.01, k3f=30, k3b=30, k4f=250, k4b=250

# ---------------------- antimycin-inhibited ---------------------

# par k0f=4, k0b=4, k1f=4, k1b=4, k5f=200, k5b=0.5, k2f=60, k2b=1, k3f=30, k3b=30, k4f=250, k4b=250

# ---------------------- myxothiazol-inhibited --------------------

# par k0f=4, k0b=4, k1f=4, k1b=4, k5f=200, k5b=0.3, k2f=0, k2b=0, k3f=30, k3b=30, k4f=250, k4b=250

# -------- stigmatellin-inhibited (parameters + initial conditions) -----------

# par k0f=4, k0b=4, k1f=4, k1b=4, k5f=200, k5b=0.3, k2f=0, k2b=0, k3f=0, k3b=0, k4f=250, k4b=250

# init QoFobCo=0,QoFobCr=0,QoFocCo=0,QoFocCr=0,QoFrbCo=0,QoFrbCr=0.5,QoFrcCo=0,QoFrcCr=0,QHoFobCo=0,QHoFobCr=0,QHoFocCo=0,QHoFocCr=0,QhoFrbCo=0,QHoFrbCr=0.5,QHoFrcCo=0,QHoFrcCr=0,EmpFobCo=0,EmpFobCr=0,EmpFocCo=0,EmpFocCr=0,EmpFrbCo=0,EmpFrbCr=0,EmpFrcCo=0,EmpFrcCr=0

# pH = 7, Eh = 100 mV

# -------------- non-inhibited cytochrome bc1 ------------------------

# par k0f=4, k0b=4, k1f=4, k1b=4, k5f=200, k5b=0.3, k2f=60, k2b=0.0, k3f=30, k3b=30, k4f=70, k4b=7000

# ---------------------- antimycin-inhibited ---------------------

# par k0f=4, k0b=4, k1f=4, k1b=4, k5f=200, k5b=0.5, k2f=60, k2b=5, k3f=30, k3b=30, k4f=9800, k4b=212

# ---------------------- myxothiazol-inhibited --------------------

# par k0f=4, k0b=4, k1f=4, k1b=4, k5f=200, k5b=0.5, k2f=0, k2b=0, k3f=30, k3b=30, k4f=1500, k4b=1500

# ------- stigmatellin-inhibited (parameters + initial conditions) -----------

# par k0f=4, k0b=4, k1f=4, k1b=4, k5f=200, k5b=0.3, k2f=0, k2b=0, k3f=0, k3b=0, k4f=1500, k4b=1500

# init QoFobCo=0,QoFobCr=0,QoFocCo=0,QoFocCr=0,QoFrbCo=0,QoFrbCr=0.5,QoFrcCo=0,QoFrcCr=0,QHoFobCo=0,QHoFobCr=0,QHoFocCo=0,QHoFocCr=0,QHoFrbCo=0,QHoFrbCr=0.5,QHoFrcCo=0,QHoFrcCr=0,EmpFobCo=0,EmpFobCr=0,EmpFocCo=0,EmpFocCr=0,EmpFrbCo=0,EmpFrbCr=0,EmpFrcCo=0,EmpFrcCr=0

########################### E Q U A T I O N S ######################

dQoFobCo/dt = k1f*Q*EmpFobCo - k1b*QoFobCo - k3f*QoFobCo + k3b*QoFocCo + k5f*CYT*QoFobCr - k5b*Cred*QoFobCo

dQoFobCr/dt = k1f*Q*EmpFobCr - k1b*QoFobCr - k3f*QoFobCr + k3b*QoFocCr - k5f*CYT*QoFobCr + k5b*Cred*QoFobCo

dQoFocCo/dt = k1f*Q*EmpFocCo - k1b*QoFocCo + k3f*QoFobCo - k3b*QoFocCo + k5f*CYT*QoFocCr - k5b*Cred*QoFocCo

dQoFocCr/dt = k1f*Q*EmpFocCr - k1b*QoFocCr + k3f*QoFobCr - k3b*QoFocCr + k4f*QoFrcCo - k4b*QoFocCr - k5f*CYT*QoFocCr + k5b*Cred*QoFocCo

dQoFrbCo/dt = k1f*Q*EmpFrbCo - k1b*QoFrbCo - k3f*QoFrbCo + k3b*QoFrcCo + k2f*QHoFobCo - k2b*QoFrbCo + k5f*CYT*QoFrbCr - k5b*Cred*QoFrbCo

dQoFrbCr/dt = k1f*Q*EmpFrbCr - k1b*QoFrbCr - k3f*QoFrbCr + k3b*QoFrcCr + k2f*QHoFobCr - k2b*QoFrbCr - k5f*CYT*QoFrbCr + k5b*Cred*QoFrbCo

dQoFrcCo/dt = k1f*Q*EmpFrcCo - k1b*QoFrcCo + k3f*QoFrbCo - k3b*QoFrcCo - k4f*QoFrcCo + k4b*QoFocCr + k5f*CYT*QoFrcCr - k5b*Cred*QoFrcCo

dQoFrcCr/dt = k1f*Q*EmpFrcCr - k1b*QoFrcCr + k3f*QoFrbCr - k3b*QoFrcCr - k5f*CYT*QoFrcCr + k5b*Cred*QoFrcCo

dQHoFobCo/dt = k0f*QH2*EmpFobCo - k0b*QHoFobCo - k3f*QHoFobCo + k3b*QHoFocCo - k2f*QHoFobCo + k2b*QoFrbCo + k5f*CYT*QHoFobCr - k5b*Cred*QHoFobCo

dQHoFobCr/dt = k0f*QH2*EmpFobCr - k0b*QHoFobCr - k3f*QHoFobCr + k3b*QHoFocCr - k2f*QHoFobCr + k2b*QoFrbCr - k5f*CYT*QHoFobCr + k5b*Cred*QHoFobCo

dQHoFocCo/dt = k0f*QH2*EmpFocCo - k0b*QHoFocCo + k3f*QHoFobCo - k3b*QHoFocCo + k5f*CYT*QHoFocCr - k5b*Cred*QHoFocCo

dQHoFocCr/dt = k0f*QH2*EmpFocCr - k0b*QHoFocCr + k3f*QHoFobCr - k3b*QHoFocCr + k4f*QHoFrcCo - k4b*QHoFocCr - k5f*CYT*QHoFocCr + k5b*Cred*QHoFocCo

dQHoFrbCo/dt = k0f*QH2*EmpFrbCo - k0b*QHoFrbCo - k3f*QHoFrbCo + k3b*QHoFrcCo + k5f*CYT*QHoFrbCr - k5b*Cred*QHoFrbCo

dQHoFrbCr/dt = k0f*QH2*EmpFrbCr - k0b*QHoFrbCr - k3f*QHoFrbCr + k3b*QHoFrcCr - k5f*CYT*QHoFrbCr + k5b*Cred*QHoFrbCo

dQHoFrcCo/dt = k0f*QH2*EmpFrcCo - k0b*QHoFrcCo + k3f*QHoFrbCo - k3b*QHoFrcCo - k4f*QHoFrcCo + k4b*QHoFocCr + k5f*CYT*QHoFrcCr - k5b*Cred*QHoFrcCo

dQHoFrcCr/dt = k0f*QH2*EmpFrcCr - k0b*QHoFrcCr + k3f*QHoFrbCr - k3b*QHoFrcCr - k5f*CYT*QHoFrcCr + k5b*Cred*QHoFrcCo

dEmpFobCo/dt = k0b*QHoFobCo + k1b*QoFobCo - k0f*QH2*EmpFobCo - k1f*Q*EmpFobCo - k3f*EmpFobCo + k3b*EmpFocCo + k5f*CYT*EmpFobCr - k5b*Cred*EmpFobCo

dEmpFobCr/dt = k0b*QHoFobCr + k1b*QoFobCr - k0f*QH2*EmpFobCr - k1f*Q*EmpFobCr - k3f*EmpFobCr + k3b*EmpFocCr - k5f*CYT*EmpFobCr + k5b*Cred*EmpFobCo

dEmpFocCo/dt = k0b*QHoFocCo + k1b*QoFocCo - k0f*QH2*EmpFocCo - k1f*Q*EmpFocCo + k3f*EmpFobCo - k3b*EmpFocCo + k5f*CYT*EmpFocCr - k5b*Cred*EmpFocCo

dEmpFocCr/dt = k0b*QHoFocCr + k1b*QoFocCr - k0f*QH2*EmpFocCr - k1f*Q*EmpFocCr + k3f*EmpFobCr - k3b*EmpFocCr + k4f*EmpFrcCo - k4b*EmpFocCr - k5f*CYT*EmpFocCr + k5b*Cred*EmpFocCo

dEmpFrbCo/dt = k0b*QHoFrbCo + k1b*QoFrbCo - k0f*QH2*EmpFrbCo - k1f*Q*EmpFrbCo - k3f*EmpFrbCo + k3b*EmpFrcCo + k5f*CYT*EmpFrbCr - k5b*Cred*EmpFrbCo

dEmpFrbCr/dt = k0b*QHoFrbCr + k1b*QoFrbCr - k0f*QH2*EmpFrbCr - k1f*Q*EmpFrbCr - k3f*EmpFrbCr + k3b*EmpFrcCr - k5f*CYT*EmpFrbCr + k5b*Cred*EmpFrbCo

dEmpFrcCo/dt = k0b*QHoFrcCo + k1b*QoFrcCo - k0f*QH2*EmpFrcCo - k1f*Q*EmpFrcCo + k3f*EmpFrbCo - k3b*EmpFrcCo - k4f*EmpFrcCo + k4b*EmpFocCr + k5f*CYT*EmpFrcCr - k5b*Cred*EmpFrcCo

dEmpFrcCr/dt = k0b*QHoFrcCr + k1b*QoFrcCr - k0f*QH2*EmpFrcCr - k1f*Q*EmpFrcCr + k3f*EmpFrbCr - k3b*EmpFrcCr - k5f*CYT*EmpFrcCr + k5b*Cred*EmpFrcCo

dQH2/dt=k0b*(QHoFobCo+QHoFobCr+QHoFocCo+QHoFocCr+QHoFrbCo+QHoFrbCr+QHoFrcCo+QHoFrcCr)-k0f*QH2*(EmpFobCo+EmpFobCr+EmpFocCo+EmpFocCr+EmpFrbCo+EmpFrbCr+EmpFrcCo+EmpFrcCr)

dQ/dt=k1b*(QoFobCo+QoFobCr+QoFocCo+QoFocCr+QoFrbCo+QoFrbCr+QoFrcCo+QoFrcCr)-k1f*Q*(EmpFobCo+EmpFobCr+EmpFocCo+EmpFocCr+EmpFrbCo+EmpFrbCr+EmpFrcCo+EmpFrcCr)

dCYT/dt = - k5f*CYT*(QoFobCr+QoFocCr+QoFrbCr+QoFrcCr+QHoFobCr+QHoFocCr+QHoFrbCr+QHoFrcCr+EmpFobCr+EmpFocCr+EmpFrbCr+EmpFrcCr)+k5b*CRED*(QoFobCo+QoFocCo+QoFrbCo+QoFrcCo+QHoFobCo+QHoFocCo+QHoFrbCo+QHoFrcCo+EmpFobCo+EmpFocCo+EmpFrbCo+EmpFrcCo)

dCRED/dt = k5f*CYT*(QoFobCr+QoFocCr+QoFrbCr+QoFrcCr+QHoFobCr+QHoFocCr+QHoFrbCr+QHoFrcCr+EmpFobCr+EmpFocCr+EmpFrbCr+EmpFrcCr)-k5b*CRED*(QoFobCo+QoFocCo+QoFrbCo+QoFrcCo+QHoFobCo+QHoFocCo+QHoFrbCo+QHoFrcCo+EmpFobCo+EmpFocCo+EmpFrbCo+EmpFrcCo)

############ P A R A M E T E R S F O R X P P A U T ##################

@ maxstor=6000000, total=200, DT=0.001, method=gear, tolerance=0.0001, maximumstep=2, minimumstep=2E-12

done
